# Assessment of feed resources, feeding practices and coping strategies to feed scarcity by smallholder urban dairy producers in Jimma town, Ethiopia

**DOI:** 10.1186/s40064-016-2417-9

**Published:** 2016-06-14

**Authors:** Belay Duguma, Geert Paul Jules Janssens

**Affiliations:** Department of Animal Sciences, College of Agriculture and Veterinary Medicine, Jimma University, P.O. Box 307, Jimma, Ethiopia; Laboratory of Animal Nutrition, Faculty of Veterinary Medicine, Ghent University, Heidestraat 19, 9820 Merelbeke, Belgium

**Keywords:** coping strategies, Dairy, Feed resources, Feeding practices, Feed scarcity, Smallholder

## Abstract

Smallholder dairy production is increasingly becoming popular in Jimma town. However, feed shortage is a major constraint to dairy production. The objectives of this study was to assess feed resources, feeding practices and farmers’ perceived causes of feed shortage and coping strategies to feed scarcity in smallholder dairy producers in Jimma town, Oromia Regional State, Ethiopia. A total of 54 randomly selected dairy farmers were interviewed using a pre-tested structured questionnaire and through direct observations. Twenty major feed types used by dairy farmers were identified and categorized into five classes: natural pasture grazing, green feeds, hay, concentrate (commercial mix and agro-industrial by-products) and non-conventional feed resources. Green feeds-fresh or succulent grasses and legumes (mean rank = 0.361), concentrate (0.256), hay (0.198), non-conventional feeds (0.115) and natural pasture grazing (0.070) were ranked as the main feed resources in that order of importance. Green feed (94.4 % of the respondents) was found to be the main basal diet of dairy cattle. Overall, wheat bran (85.2 % of the respondents), commercial concentrate (55.6 %), noug (*Guizotia abyssinica*) cake (20.4 %), cotton seed cake (7.4 %) and molasses (7.4 %) were the main concentrate supplements used (P > 0.05). Local brew waste (*attela*) (77.8 % of the respondents), bean and pea hulls (42.6 %) enset (*Ensete ventricosum*) leaf and pseudo-stem (37 %), sugarcane tops (33.3 %), banana leaf and stem/stover (16.7 %) and papaya stem (16.7 %) were the dominant non-conventional feed resources in the surveyed area (P > 0.05). About 79.6, 7.4, 1.9 and 11.1 % of the farmers used zero-, semi-zero-, and the combination of zero- and semi-zero- and free-grazing systems, respectively. Most farmers (90.7 %) offered concentrate supplements to milking cows. However, supplementation did not consider milk yield, physiological status and condition of cows. All the farmers (100 %) offered common salt to their cattle as mineral supplement. The majority (98.1 %) of the farmers experience feed shortage in the dry season. Land scarcity (55.6 % of the respondents) was reported as the most important cause of feed scarcity followed by a combination of land scarcity and poor feed availability (42.2 %). Increasing use of agro-industrial by-products and commercial concentrate mix (87 % of the respondents), increasing use of hay (74.1 %), increasing use of non-conventional feeds (50 %), purchasing green feeds (19.8 %) and reducing herd size (2.7 %) were the strategies adopted for coping with feed scarcity. From results of this study, it could be concluded that to ensure sustainable availability of dairy cattle feed in the surveyed area, technological, technical and institutional innovations would be vital.

## Background

In Ethiopia, agriculture is the main economic activity with more than 80 % of the population being dependent on this practice in which livestock play a very important role (CSA 2009). Agriculture contributes with approximately 50 % to the overall gross domestic product (GDP), generates 90 % of export earnings and provides employment for 80 % of the population (CSA 2009). Livestock is a crucial part of the agriculture and the contribution of livestock and their products to the agriculture economy accounts for 47 % (IGAD 2011).

Ethiopia holds the largest cattle population in Africa with an estimated herd of approximately 53.4 million head (CSA 2011). There are 10 million indigenous dairy cows yielding 3.2 billion liters of milk per year, with national average milk production of 1.54 L per cow per day (CSA 2008).

Despite the large dairy cattle population, milk production per cow per day is very low in Ethiopia. The low productivity is principally due to inefficient nutritional and management practices, low genetic potential of the indigenous cows, high level of disease and parasitic incidence, poor access to extension and credit services, and inadequate information to improve animal performance (Getahun 2012; Aynalem et al. 2011; Zegeye 2003). Among these constraints, inadequate quantity and quality feed ingredients were identified as a major limiting factor to the development of dairy production in peri-urban and urban dairy systems (Belachew et al. 1994; Staal and Shapiro 1996; Zelalem 1999; Yitaye et al. 2008; Zegeye 2003; Teferee 2003; Asaminew and Eyasu 2009; Belay et al. 2011).

Livestock feed resources in Ethiopia are mainly obtained from natural and improved pastures, crop residues, forage crops, agro-industrial by-products and non-conventional feeds (CSA 2012). The contribution of these feed resources, however, depends up on the agro-ecology, the type of crop produced, accessibility and production system (Seyoum et al. 2001; Ahmed et al. 2010). Natural pasture is the major source of livestock feed in Ethiopia. However, its importance is gradually declining because of the expansion of crop production into grazing lands, redistribution of common lands to the landless and land degradation (Berhanu et al. 2009). Urban and peri-urban dairy production depends on purchased concentrate and roughage feeds with limited grazing (Azage et al. 2013).

The Ethiopian government has given strong attention to the development of smallholder market-oriented dairy production in peri-urban and urban production systems to increase milk production, including Jimma town area. These production systems have a tremendous potential for development and could play a significant role in minimizing the acute shortage of dairy products in Jimma town area (Azage et al. 2000).

In the present study area, small-scale market-oriented urban dairy production is increasingly becoming popular for income generation, family nutrition and employment. This dairy system is contributing immensely towards filling in the large demand–supply gap for milk and milk-based products, caused by population, income and urban growths. However, farmers in the study area ranked feed shortage as their first major constraint to dairy production (Belay et al. 2011).

Understanding the various feed resources and coping strategies used by farmers to overcome feed shortage is important in order to identify appropriate research and development interventions to enhance health and performance of dairy cattle. However, there is no such work done in Jimma town to plan technical and institutional interventions. The aim of this study was to identify the available feed resources, feeding systems and farmers’ coping strategies with feed scarcity under smallholder urban dairy system in Jimma town, Oromia Regional State, Ethiopia.

## Methods

### Description of study area

This study was conducted in Jimma, capital town of Jimma Zone of Oromia Regional State, Ethiopia. Jimma is 352 km from Addis Ababa, capital city of Ethiopia and is located at 7°4′N latitude and 36°50′E longitude and situated at an altitude of 1704 m above sea level. The area has sub-humid tropical climate with average annual rainfall ranging from 1200 to 2000 mm, having a bimodal pattern. Approximately 70 % of the total annual rainfall is received during the main rainy season, which lasts from June to September. The short rainy season extends from March to May. The dry season lasts from October to February. The average annual minimum and maximum temperatures are 25 to 30 °C, respectively (Statistical abstract 2002).

### Sampling procedure

The target population for this study was the entire list of households in Jimma town keeping dairy cattle at the time of this study. A list of 78 smallholder dairy cattle farmers in the town was obtained from records maintained by the Jimma Town Multipurpose Dairy Development Private Limited Company and Jimma Town Administration Agricultural Development Office. The number of households sampled in the study area was determined by N = 0.25/SE^2^, where N = number of sampled households, SE = standard error (Arsham 2005). Considering standard error of 0.068 with 95 % coefficient interval as follows, N = 0.25/(0.068)^2^ = 54. Then a total of 54 smallholder dairy farmers were randomly selected. The randomly selected farms/households were stratified again into three groups based on the number of dairy cattle owned as: small farms owning 1–9 dairy cattle (18 farms), medium farms 10–18 animals (18 farms) and large farms with more than 19 animals (18 farms). In total, 54 households (69 % of 78 households) were randomly selected for the study. Before the formal survey, a preliminary visit was made by the first author and animal health assistant of the dairy cooperative to get the consent of the farmers, locate the farms and to give a brief description to each respondent on our research objectives and potential benefit of involving in the study.

### Data collection

A single-visit-multiple-subjects formal survey technique (ILCA 1990) was used to collect data through household interviews, conducted in the local languages by the researcher using a pre-tested, structured questionnaires and personal observation. The questionnaire was prepared in English, and translated into the local ‘Afaan Oromo’ and ‘Amharic’ languages by the first author of the current study, who is a fluent in both local languages. The data collected included: socio-economic characteristics of the respondents, conventional and non-conventional feed resources, feeding practices/system, sources of feed acquisition, ranking of feed resources, practice of feed supplementation, farmers’ perception of feed shortage and coping strategies to feed scarcity, practice of improved forage production and monthly feed expenses.

### Statistical analysis

The computer software Excel was used for data management and entry. All the collected survey data were coded and entered into the computer with Excel. The Statistical Package for Social Sciences (SPSS) software (version 16.0) computer programs was used for data analysis. The analysis included descriptive statistics (means, frequencies and percentages). Indices (weighted averages) were developed to obtain the aggregate ranking of the major feed resources utilized in the study area and calculated as: Index = sum of [(4 × number of responses for 1st rank + 3 × number of responses for 2nd rank + 2 × number of responses for 3rd rank + 1 × number of responses for 4th)]/(4 × total responses for 1st rank + 3 × total responses for 2nd rank + 2 × total responses for 3rd rank + 1 × total responses for 4th rank).

## Results and discussion

### Household characteristics

Household characteristics of the respondents in the study area are shown in Table 1. Overall, mean age of the household heads was found to be 51.26 ± 10.99 years and there was no difference (P > 0.05) among farm sizes. The result shows that farmers with old age were involved in dairy production in Jimma town. The overall mean family size was 6.02 ± 2.52 persons/household and there was no difference (P > 0.05) among the farm sizes. This result is lower than the findings of Haile et al. (2012) for Hawassa town (7.1 ± 0.22 persons), Asaminew and Eyasu (2009) for Bahir Dar Zuria (8.2) and Mecha woredas (7.2). Large family size was considered very important for dairy activities. In this study about 17 and 50 % of the respondents used family labour and a combination of family and hired labour, respectively (Belay et al. 2011).

**Table 1:** Household characteristics of smallholder dairy farmers in Jimma town

Variables	Farm type				
	Small (n = 18)	Medium (n = 18)	Large (n = 18)	Total	P value
Age [mean (±SE)]	50.06 ± 2.63^a^	50.94 ± 2.98^a^	52.78 ± 2.22^a^	51.26 ± 1.50	0.758
Family size [mean (±SE)]	5.28 ± 0.39^a^	6.17 ± 0.70^a^	6.61 ± 0.63^a^	6.02 ± 0.34	0.276
Sex (%)					0.667
Male	24.1	24.1	27.8	75.9	
Female	9.3	9.3	5.6	24.1	
Occupation (%)					0.528
Trade/business	5.6	3.7	11.1	20.4	
Civil servant	7.4	11.1	7.4	25.9	
Retirement	7.4	9.3	7.4	25.9	
Dairy farmer	5.6	5.6	5.6	16.7	
Housewife	7.4	3.7	0	11.1	
Education (%)					0.163
Illiterate	0	1.9	0	1.9	
Primary school	3.7	11.1	5.6	20.4	
Junior secondary	7.4	1.9	1.9	11.14	
Senior secondary	13.0	5.6	5.6	24.1	
College	5.6	11.1	18.5	35.2	
University	3.7	1.9	1.9	7.4	

Means with the same superscript letters in the same row are not significantly different

Overall, 75.9 and 24.1 % of the farmers were male and female-headed households, respectively, and there was no difference (P > 0.05) among farm sizes. The proportion of female-headed households in the present study was lower than the 47.7 % for Hawassa town (Haile et al. 2012) and the 33 % for Addis Ababa (Azage 2004). Our result was also in agreement with the findings of previous studies by (Teferee 2003; Azage 2004) for Addis Ababa and Yitaye et al. (2008) in northwest Ethiopia, who reported most of the urban dairy farmers were male-headed. In this study, the proportions of female-headed households were relatively few (5.6 %) in large farm sizes.

Our results revealed that, 25.9 % of the dairy farmers were civil servants, while traders, full-time dairy farmers, retired employees and housewives were 20.4, 16.7, 25.9 and 11.1 %, respectively. There were more traders in large sized farms (11.1 %) and more public servants (11.1 %) in medium sized farms. Results of the current study revealed that the majority of the respondents had different off-farm economic activities and took dairying as a side business to supplement their income. Smith and Hogan (1999) reported that urban dairy farming can be a part-time activity where household members work in other sectors of the urban economy.

About 20.4, 11.1, 21.4, 35.5 and 7.4 % of the interviewed farmers had primary, junior secondary, senior secondary, college and university education, respectively and there was no variation (P > 0.05) among the farm sizes. The majority of the dairy farmers obtained their diploma in general agriculture from College of Agriculture of Jimma University located in Jimma town. The proportion of dairy farmers who had college and university education in the current study was higher than the result of Yousuf (2003) who reported 24 % for Harar town (Ethiopia). Majority of the respondents in the present study had formal education and is important to understand extension messages and to realize the importance of new technologies within a short time. According to Ofukou et al. (2009) farmers with high educational levels usually adopt new technologies more rapidly than lower educated farmers.

### Feed resources

The major feed resources in the study area are shown in Table 2. In the present study, twenty different feed resources were identified and categorized into: natural pasture grazing, hay, green feeds (fresh or succulent grasses and legumes), concentrates (noug cake, cotton seed cake, grains, molasses, wheat bran, commercial concentrate mix and brewery grain waste and non-conventional feed resources (banana leaves and stems, enset (*Ensete ventricosum*) leaves and pseudo-stems, papaya stems, *attela* (a by-product of local alcoholic brew), Khat (*Catha edulis*) leaves, bean and pea hulls and grain wastes (*bulule*). *Bulule* is the waste of the mixture of cereals grain (*tef*, wheat, barley, maize, oats etc.) of mill houses. The finding of the present study with regard to identified feed resources was in agreement with previous works (Yoseph 1999; Yosef et al. 2003; Nigussie 2006; Adugna 2008; Mureda and Zeleke 2008; Sintayehu et al. 2008; Yitaye et al. 2008), who reported natural pasture, hay, agro-industrial by-products, commercial concentrate and non-conventional feeds were the most important feed resources used by urban dairy producers in different parts of Ethiopia.

**Table 2:** Frequencies (%) of feed resources as reported by smallholder dairy farmers in Jimma town

Feed resources	Type of farm				
	Small (n = 18)	Medium (n = 18)	Large (n = 18)	Total	P value
Natural pasture grazing	11.1	5.6	7.4	21.1	0.492
Hay	22.2	25.9	31.5	79.6	0.114
Green feed	29.6	31.5	33.3	94.4	0.347
Cereal grains	0	0	1.9	1.9	0.361
Wheat bran	27.8	31.5	25.9	85.2	0.358
Commercial concentrate	13.0	22.2	20.4	55.6	0.207
Molasses	3.7	3.7	0	7.4	0.340
Brewery spent grain	0	3.7	1.9	5.6	0.347
Noug cake	9.3	3.7	7.4	20.4	0.450
Cotton seed cake	1.9	1.9	3.7	7.4	0.763
Maize powder	0	0	3.7	3.7	0.125
Local brewery by-product (*Attela*)	27.8	25.9	24.1	77.8	0.725
Khat (*Catha edulis*) leaf	0	3.7	0	3.7	0.125
Banana leaves and pseudo-stems	11.1	1.9	3.7	16.7	0.061
Enset leaves and pseudo-stems	13.0	14.8	9.3	37	0.574
Papaya stems	7.4	3.7	5.6	16.7	0.670
Sugar cane tops	11.1	13.0	9.3	33.3	0.779
Bean and pea hulls	16.7	13.0	13.0	42.6	0.739
Mill house waste (*bulule*)	7.4	0	0	7.4	0.013
Tree legumes	0	0	1.9	1.9	0.361
Common salt	33.3	33.3	33.3	100	

Regardless of farm size, the majority (94.4 %) of interviewed farmers used green feeds as the main basal diet, especially during the wet seasons. Green feeds were mainly available from June to September (wet season) for purchase and from open areas for free. About 29.6 % small, 31.5 % medium and 33.3 % large size farms used green feeds (P > 0.05).

Wheat bran (85.2 % of the respondents), commercial concentrate mix (55.6 %) and noug (*Guizotia abyssinica*) cake (20.4 %) were the most important concentrate supplements, with minor use of others, such as molasses, cotton seed cake and maize powder. Wheat bran was found to be utilized by all farm groups, 27.8 % small, 31.5 medium and 25.9 % large size farms. Wheat bran was the predominantly used agro-industrial by-product due to its high accessibility and relatively low cost obtained at dairy cooperative and retailers. Commercial concentrate mix was observed to be frequently used by medium (22.2 %) and large (20.4 %) sized farms, for which it is available at affordable price.

It was observed that dairy farmers in the studied area did not restrict themselves to the use of conventional feed resources only, but made use of locally available non-conventional feed resources, particularly during the dry seasons. Banana leaves and pseudo-stems (11.1 %), bean and pea hulls (16.7 %) and *bulule* (7.4 %) were utilized most by farmers from small farm size, who cannot afford to purchase agro-industrial by-products and concentrate mix due to financial limitations.

Overall, local brew wastes (*attela*), bean and pea hulls, enset leaves and pseudo and stem tubers and sugar cane tops were used by 77.8, 42.6, 37 and 33.3 % of the respondents, respectively. The wide utilization of *attela* was due to its availability throughout the year from local brews with low cost. Overall, few farmers (22.1 %) relied on natural grazing lands. In the present study, all the respondents (100 %) stated that they provided common salt as mineral supplement to all classes of dairy cattle, and commercial mineral supplementation is non-existent. This is in line with the findings of Haile et al. (2012) who reported that urban dairy farmers in Hawassa city (Ethiopia) supplement their animals with common salt.

The respondents reported that despite increased milk production from feeding papaya, enset and banana stems, they affect the quality of milk negatively by increased water content, which in turn raised complaint from consumers. This was in agreement with earlier reports of Fekadu and Ledin (1997). *Bulule*, a waste of the mixture of cereal grains (*tef*, wheat, barley, maize, oats etc.) powder of mill houses was used only by small size farms (P < 0.05) due to its low price. The respondents stated that feeding *bulule* has improved milk production of dairy cows. The reason for the increase could be due to the high energy and protein content of the different cereal grains and pulses in the mill house wastes. In this study, utilization of tree legumes and other cultivated forages was not well adopted.

In general, in the present study, the availability of feed resources varied across seasons, and farmers utilized whatever is available for feeding dairy cattle. During the wet season, concentrate supplements and green feeds are the most widely used feed resources, whereas during the dry season the poor quality natural pasture for those who grazed their animals, conserved hay, concentrates, non-conventional feeds are important feed resources. According to the respondents, this variation in seasonal feed availability and quality resulted in low milk production and low income. Thus, it is important to effectively utilize the available feed resources and provide concentrate supplements to alleviate the feed shortage and maximize milk production of dairy cows.

### Feeding system

In the present study, three types of dairy feeding systems were practiced: zero-grazing/stall feeding (79.6 %), zero- and partial-grazing (7.4 %) and full time-grazing (11.1 %). The dairy animals are managed indoors and farmers used cut-and-carry feeding systems. Most farmers in large size farms (31.5 %) practiced zero-grazing as compared to small sized farms (20.4 %) and medium sized farms (27.8 %) (P > 0.05). However, high proportion of farmers in the small size farms (11.1 %) practiced free-grazing. This could be due to lack of capital to purchase and feed concentrate feeds. In agreement with the current findings, 72 % of the smallholder dairy farmers in Dire Dawa town practiced zero-grazing system (Mureda and Zeleke 2008). In this study, majority of the farmers practiced dairy farming in the family residential compounds. The adoption of zero-grazing by majority of the respondents addresses the problem of access to land for feed production.

The result of the current study revealed that about 81.1 % of the respondents used purchased feed. This is in agreement with the findings of Sintayehu et al. (2008), who reported that 76 % of urban dairy farmers in southern Ethiopia used purchased feeds. Our finding revealed that the primary reason for the predominant use of purchased feeds is attributed to scarcity of land for on farm forge cultivation (Table 3).

**Table 3:** Feeding practices and acquisition of concentrate feeds by dairy farmers in Jimma town

Variable	Type of farm				
	Small	Medium	Large	Total	P value
Grazing system					0.253
Zero-grazing	20.4	27.8	31.5	79.6	
Semi-grazing	3.7	1.9	1.9	7.4	
Zero- and full grazing	1.9	0	0	1.9	
Free grazing	7.4	3.4	0	11.1	
Sources of feed acquisition					
Purchased	24.1	27.8	29.6	81.5	
On farm and purchased	0	1.9	3.7	5.6	
Communal pasture grazing	1.9	1.9	0	3.7	
Purchased and grazing	7.4	1.9	0	9.3	

### Farmers’ perceived ranking of feed resources

Table 4 shows farmers’ ranking of feed resources by the dairy farmers in the study area. The respondents were asked to rank the identified major feed resources for feeding dairy cattle. The respondents prioritized the identified feed resources according to their perceived importance. Green feeds (mean rank = 0.361), concentrate feeds (0.256), hay (0.198), non-conventional feeds (0.115) and natural grazing land (0.070) were ranked as the main feed resources. Green grasses and legumes of native species common found in the area were ranked as the first most important feed resources. Green feeds were widely utilized as basal diet during the rainy seasons. In the wet seasons, they are readily available and purchased from compounds of government offices, schools, and military camps and from individuals. Sometimes they can be collected from open access areas, in forests, road and river sides for free for cut-and-carry feeding. Concentrate supplements were ranked as the second most important feed resources, and used year round for supplementing dairy cows at milking. They are purchased from feed industries, retailers and from the dairy cooperative, which supplies most of the concentrate supplements to its members and non-members. Respondents ranked conserved hay of native grasses and legumes as the third most important feed resource in the dry seasons. Farmers make hay from the same sources as green feeds. Natural grazing lands had the lowest ranking within the common feed resources ranked by the farmers. It was due to the zero-grazing system practiced by the majority of dairy farmers in the current studied area.

**Table 4:** Major feed resources as ranked by smallholder dairy farmers in Jimma town

Feed resources	Ranked (no. of responses)				
	Rank 1	Rank 2	Rank 3	Rank 4	(Rank) mean index^a^
Grazing	9	–	1	–	(5) 0.070
Hay	2	9	35	2	(3) 0.198
Green feed	40	7	5	4	(1) 0.361
Concentrate^b^	3	36	9	–	(2) 0.256
Non-conventional feeds	–	2	4	48	(4) 0.115
Total	54	54	54	54	1.00

Index = [(4 × number of responses for 1st rank + 3 × number of responses for 2nd rank + 2 × number of responses for 3rd rank + 1 × number of responses for 4th)] divided by (4 × total responses for 1st rank + 3 × total responses for 2nd rank + 2 × total responses for 3rd rank + 1 × total responses for 4th rank)

^a^The higher the rank for a given reason, the greater its importance

^b^Includes agro-industrial by products and commercial concentrate

### Practice of concentrate supplementation

Table 5 shows the frequency of households allocating supplementary feeds to their cattle. Ninety-eight percent of the respondents supplement concentrate feeds. Based on the economic importance of the class of cattle, concentrate supplementation varied among classes of cattle kept. Overall, the majority of the farmers (94.4 %) offered concentrate supplements mainly to milking cows (P > 0.05), without considering milk yield, physiological status and body condition of the cows. Thus, this study suggests that farmers should be advised to consider milk yield and physiological stage and body condition in their future supplementation of milking cows, in order to increase their profit from milk production.

**Table 5:** Practices of feed supplementation (%) by dairy farmers in Jimma town

Variable	Type of farm				
	Small	Medium	Large	Total	P value
Practice of concentrate supplementation					0.361
Yes	33.3	31.5	33.3	98.1	
No	0	1.9	0	1.9	
Types of supplements used					
Noug cake	5.6	3.7	3.7	13.0	0.182
Concentrate mix	0	3.7	0	3.7	
Wheat bran and concentrate mix	20.4	22.2	29.6	72.2	
Bean and pea hulls	3.7	3.7	0	7.4	
Wheat bran and hulls	3.7	3.7	0	7.4	
Class of animals supplemented					0.191
Milking cows	33.3	31.5	29.6	94.4	
Milking cows and calves	0	1.9	0	1.9	
All classes of cattle	0	0	3.7	3.7	

The main reason for supplementing mostly milking cows was to maximize milk production. The results of our study was in line with the findings of Sintayehu et al. (2008) who reported that 58 % of the farmers provided supplementary feeds mainly to lactating cows. This study suggests the need for offering concentrate supplements to replacement heifers in order to attain early age at puberty and first calving. Azage and Alemu (1998) have indicated that dietary supplementation of heifers during their growth will reduce the interval from birth to first calving; probably because heifers that grow faster will cycle earlier and allow easier estrus detection.

Overall, 70.4 % of the farmers, 20.4 % from small, 22.2 % from medium and 29.6 % from large farms used the combination of wheat bran and commercial concentrate mix followed by wheat bran alone (13 %) as concentrate supplements. Those farmers who could not afford to buy wheat bran and concentrate mix used mill house wastes (7.4 %) to supplement their milking cows. The respondents reported that they were well aware of the high benefits of using concentrate supplements in increasing milk production, however, lack of capital, low availability, high cost, problem of transportation and storage facilities were reported to be the main limitations to their adequate utilization. The distance to the sources of most concentrate supplements was also among the constraints to their use. For instance, Addis Ababa, where most concentrate feeds are obtained is located at distance of 255 km.

All the interviewees reported that they also supplement dairy cattle with non-conventional feeds, particularly in the dry seasons. Even though non-conventional feeds are not yet widely recognized by some farmers, they have high potential to be utilized during the dry seasons. Thus, adequate training and extension services on the potential importance of non-conventional feed resources available in the surveyed area would be important to alleviate feed shortage and cost.

### Farmers’ coping strategies to feed scarcity

Farmers’ coping strategies to feed shortage in the study area is presented in Table 6. Feed shortage, especially during the dry season is the main problem of the smallholder dairy producers in urban areas. Majority (98 %) of the respondents reported that they experience feed shortages in the dry seasons (P > 0.05), mainly because of land scarcity (55.6 %) followed by a combination of land shortage and poor feed availability (42.4 %). The causes of feed shortage were similar across the farm sizes studied. Lack of access to land was stated by the respondents as the most important cause of feed availability. During the dry seasons, there is shortage of green feeds which were widely used as basal diet during the rainy season. The low availability and quality of feeds in the dry seasons tends to affect the productive and reproductive performance of dairy cows unless the animals are adequately supplemented. According to Tsehay (1999), poor nutrition increases the susceptibility of dairy cows to health problems and physiological stress which results in lower production, much longer calving interval, as well as problem in fertility.

**Table 6:** Farmers perceived feed shortage, possible causes and coping strategies in the study area (%)

Variable	Farm type				
	Small	Medium	Large	Total	P value
Do you experience feed shortage in the dry season, yes	33.3	31.5	33.3	98.1	0.361
Perceived causes of feed shortage in the dry season					0.207
Shortage of land	24.1	16.7	14.8	55.6	
Poor availability of feeds	9.3	16.7	18.4	44.4	
Adopted coping strategies to feed scarcity in the dry season					
Increased use of concentrate feeds	27.8	29.6	29.6	87	0.849
Purchase green feeds	5.6	1.9	7.4	14.8	0.358
Increased use of hay	25.8	22.2	24.1	74.1	0.509
Reduce herd size	0	1.9	1.9	3.8	0.595
Use non-conventional feeds	18.9	18.9	11.3	49.1	0.387
Feed shortage in the rainy season, yes	1.9	1.9	0	3.7	0.595
Causes of feed shortage in the rainy season					
Water logging on natural grazing lands	1.9	1.9	0	3.7	0.595
Coping strategies to feed scarcity in rainy season					
Increased use of concentrates and hay	1.9	1.9	0	3.7	0.361

Farmers’ adopted coping strategies with dry season feed scarcity were increasing use of agro-industrial by-products and concentrate mix (87 %), increasing use of conserved hay (74.13 %), increasing use of non-conventional feeds (50 %), purchasing green feeds when available (14.8 %) and reducing herd size (3.7 %) and showed no significant difference (P > 0.05) among farm sizes. Jayasuriya (2002) reported that when smallholder farmers in developing countries faced with limited feed availability for feeding livestock they use what is locally available to them, at either no or low costs. The majority (96.3 %) of the respondents reported that they did not experience feed shortage during the wet season. In contrary, Asaminew and Eyasu (2009) reported that farmers in the northeast Ethiopia experience feed shortage both in dry and wet seasons.

Based on the results of our study, feed shortage was stated as a major problem to increase milk production, especially in the dry seasons. This suggests the need for a government intervention and allocating to the dairy farmers for feed production. The introduction of urea molasses mineral blocks, fodder conservation practices particularly hay making and effective utilization of the locally available crop residues would be crucial in order to enable sustainable feed availability throughout the year.

### Practice of improved forage production

Table 7 represents the practice of improved forage production in the study area. Ninety-eight percent of the respondents reported that they did not practice improved forage production. In, contrary, 58 and 67 % of dairy farmers in Nekemte and Bako towns in western Oromia practiced improved forage production (Diriba et al. 2012). In the current study, the major limitations for not growing improved forages were land scarcity (92.6 %), both land scarcity and lack of knowledge (1.9 %), lack of awareness (3.7 %) and lack of input supply and labour shortage (1.9), respectively. Shortage of land was reported as the most limiting factor to urban dairy production (Belay et al. 2011; Azage et al. 2013).

**Table 7:** Frequency of practices of improved forage production by dairy farmers in Jimma town

Variable	Farm type				
	Small	Medium	Large	Total	P value
Improved forage production					0.361
Yes	0	0	1.9	1.9	
No	33.3	33.3	31.5	98.1	
Reasons for not growing					0.227
Shortage of land	33.3	29.6	29.6	92.6	
Lack of awareness	0	3.7	0	3.7	
Lack of land and awareness	0	0	1.9	1.9	
Lack of input and labour shortage	0	0	1.9	1.9	

Our personal observations showed that dairy farmers in the surveyed area could grow multipurpose legume trees, such as Leucaena and Sesbania as live fence. These feeds are good sources of protein and minerals for dry season feeding. However, farmers lack knowledge on the importance of these tree legumes which are commonly available among the coffee growing farmers in the rural areas. Napier or elephant grass was distributed to the rural mixed crop-livestock farmers by Agricultural Development Office of Jimma Zone and the cuttings can also be obtained from College of Agriculture and Veterinary Medicine of Jimma University. Napier grass has a good potential to increase dairy cattle feed availability and is well adapted to the local climate, give high yield and requires small area and knowledge of propagation. However, the extension service of the Urban Agriculture Office of the Jimma Town was found to be very weak to contact and train dairy farmers on improved forage production, conservation and utilization.

### Monthly feed costs

A monthly feed cost in the study area is shown in Table 8. The main component of the operating cost of dairy farming is the feed cost. On average, respondents in the study area spent 1914.26 ± 209.04 Ethiopia birr (ETB) per month, ranging from 50 to 7000 ETB for purchasing roughage and concentrate feeds. The present study revealed that large size farms invested more to purchase feeds every month than small and medium size farms (P < 0.05). This is due to the holding of large number of dairy animals by large size dairy farms and more income from sell of milk to purchase feeds. The farmers reported that the feed cost is the major financial expenditure of the total cost of dairy production. This agrees with results of Belachew et al. (1994) who reported that the proportion of feed cost to total production cost of a dairy farm is higher than other cost components.

**Table 8:** Mean (±SE) of monthly feed expenses of dairy farmers in Jimma town

Farm type	Feed cost per month per farm			
	Mean ± SE	Minimum	Maximum	P value
Small	984.44 ± 171.40^a^	120	2500	0.000
Medium	1473.89 ± 264.24^a^	50	3500	
Large	3284.44 ± 369.89^b^	320	7000	
Overall	1914.26 ± 209.04	50	7000	

Means within a column with different superscript letters are significantly different (P < 0.05)

## Conclusions

The current study has shown that Green feed, concentrates, hay, non-conventional feeds and natural pasture grazing, in that order, were the most important feed resources in Jimma town. Wheat bran and commercial concentrate mix were the dominant concentrates for supplementation. Feed scarcity was identified as the most important constraint especially during the dry season. Lack of access to land was reported as the most important cause of feed scarcity. Farmers’ adopted coping strategies against feed shortage were increased utilization of agro-industrial by-products and commercial concentrate mix, conserved hay, non-conventional feeds, purchased green feeds and reducing herd size. It is concluded that to ensure sustainable availability of dairy cattle feed in the surveyed area, technological, technical and institutional innovations would be vital. Government intervention in allocating land to the smallholder dairy farmers for feed production should be the first important intervention. The adoption of tree legume forages as live fences, use of Urea-Molasses-Blocks and increased conservation and proper storage of hay, and utilization of the locally available crop residues could be of importance in alleviating feed shortage and reducing the high feed costs.
